# *Plasmodium falciparum* Metacaspase PfMCA-1 Triggers a z-VAD-fmk Inhibitable Protease to Promote Cell Death

**DOI:** 10.1371/journal.pone.0023867

**Published:** 2011-08-17

**Authors:** Benoît Meslin, Abdoul H. Beavogui, Nicolas Fasel, Stéphane Picot

**Affiliations:** 1 Malaria Research Unit, ICBMS UMR 5246 CNRS-UCBL1-INSA, Lyon, France; 2 Department of Biochemistry, University of Lausanne, Epalinges, Switzerland; Louisiana State University, United States of America

## Abstract

Activation of proteolytic cell death pathways may circumvent drug resistance in deadly protozoan parasites such as *Plasmodium falciparum* and *Leishmania*. To this end, it is important to define the cell death pathway(s) in parasites and thus characterize proteases such as metacaspases (MCA), which have been reported to induce cell death in plants and *Leishmania* parasites. We, therefore, investigated whether the cell death function of MCA is conserved in different protozoan parasite species such as *Plasmodium falciparum* and *Leishmania major*, focusing on the substrate specificity and functional role in cell survival as compared to *Saccharomyces cerevisae*. Our results show that, similarly to *Leishmania*, *Plasmodium* MCA exhibits a calcium-dependent, arginine-specific protease activity and its expression in yeast induced growth inhibition as well as an 82% increase in cell death under oxidative stress, a situation encountered by parasites during the host or when exposed to drugs such as artemisins. Furthermore, we show that MCA cell death pathways in both *Plasmodium* and *Leishmania,* involve a z-VAD-fmk inhibitable protease. Our data provide evidence that MCA from both *Leishmania* and *Plasmodium falciparum* is able to induce cell death in stress conditions, where it specifically activates a downstream enzyme as part of a cell death pathway. This enzymatic activity is also induced by the antimalarial drug chloroquine in erythrocytic stages of *Plasmodium falciparum*. Interestingly, we found that blocking parasite cell death influences their drug sensitivity, a result which could be used to create therapeutic strategies that by-pass drug resistance mechanisms by acting directly on the innate pathways of protozoan cell death.

## Introduction

A better understanding of the mechanisms involved in protozoan cell death could provide an opportunity to design parasite-specific pro-apoptotic drugs in the aim of controlling parasitic disease.

Although protozoan parasites exhibit most of the cellular and molecular markers described in higher eukaryotes [1], information on molecular pathways involved in protozoan cell death (CD) is still limited [2]. It is known, however, that the metazoan caspase genes encoding major CD effector proteases are absent in prokaryotes, plants, fungi and protozoan parasites such as *Leishmania* and *Plasmodium* [3], [4]. *In silico* studies of these organisms have revealed the presence of metacaspases (MCA) with a C14 domain harbouring a cysteine and histidine caspase-like catalytic dyad [5], [6]. The structural homology of the catalytic domains between metacaspase and caspase suggests that metacaspase could be involved in CD through an apoptotic-like pathway [7], [8], [9]. Indeed, its activity requires a functional catalytic domain generated by autoprocessing, which is reminiscent of some caspase activation [10], [11], [12], [13]. Recently, the importance of the CD effector function of metacaspase has been supported by experimental evidence in *Arabidopsis thaliana* (*A. thaliana*) and in *Leishmania major* (*L. major*) [14], [15].

While a single metacaspase (LmjMCA) with the expected catalytic amino acids is encoded in the genome of *L. major* (*LmjF35.1580*), three metacaspase genes are annotated in the *Plasmodium falciparum* (*P. falciparum*) genome, PfMCA1 (PF13_0289), PfMCA2 (PF14_0363) and PfMCA3 (PF14_0160). PfMCA1 is the only *P. falciparum* metacaspase presenting the required histidine and cysteine catalytic dyad [13]. Interestingly, when PfMCA1 is expressed *in vitro* or in COS7 cells, it undergoes autoprocessing, which removes the prodomain [13]. This processing of PfMCA1 and the release of PfMCA1 catalytic domain is likely to be important for its CD function in *P. falciparum* as was similarly demonstrated with *A. thaliana* MCA (AtMCA) and LmjMCA [14], [15].

The single yeast metacaspase (YCA1) of *Saccharomyces cerevisae* (*S. cerevisae*) is involved in stress-induced apoptosis instigated by oxidation, acidification or aging [10], [16], [17]. The importance of YCA1 activity has been disputed by a subsequent study [18]; a discrepancy that perhaps highlights the pathway's sensitivity to specific experimental conditions of CD induction. *S. cerevisiae* is a useful model system to study metacaspase function [10] in which the MCA of various protozoa have been expressed with the purpose of elucidating their functional phenotype and effector molecules. The expression of *Trypanosoma brucei* (*T. brucei*) metacaspase 4 (TbMCA4) in yeast exhibits a phenotype of mild respiratory deficiency [19] while AtMCA and LmjMCA expression are known to induce death in budding yeasts [11], [12]. The MCA cysteine protease activity is known to have an arginine/lysine specificity [11], [12], [18], therefore, excluding caspase-like substrates. This increased affinity towards arginine substrates correlates with enzymatic processing of the precursor polypeptide in an active catalytic domain [12].

Although most protozoan parasites do not encode caspase(s), CD in protozoan parasites make use of pan-caspase substrates or inhibitors such as carbobenzoxy-valyl-alanyl-aspartyl-[O-methyl]-fluoromethylketone (z-VAD-fmk), supporting the hypothesis that proteases other than metacaspases act in the CD pathway of protozoan parasites. As recently shown in *P. falciparum* [20], the CD pathway could be mediated by activation of clan CA cysteine proteases, such as cathepsins and calpains, linked to downstream mitochondrial outer membrane permeabilization (MOMP) as well as the release of CA protease amplified by mitochondrial dysfunction and DNA degradation. Z-VAD-fmk is often used as a phenotypic marker of CD and known to block it in several unicellular organisms such as *Dictyostelium* and yeast [21], [22]. In *Leishmania*, cathepsin-B-like is accountable for the binding of z-VAD-fmk and is implicated in CD [23]. Thus far, it is not known whether metacaspase and CA protease(s) are part of the same pathway, or whether their actions are complementarily linked, leading to MOMP, reactive oxygen species production, cytochrome c release and DNA fragmentation.

Parasites encounter oxidative stress when exposed to antiparasitic drugs such as antimony or chloroquine [24], [25], [26], in the insect vector [27], [28], when entering the host cell [29], [30], [31], [32] or in the host [33], [34]. In this study, using complemented metacaspase-deficient yeast cells (Δ*yca1*), we compared the catalytic domain function of the PfMCA1 (PfMCA1-cd-Sc), YCA1 and *L. major* catalytic domain (LmjMCA-cd) under oxidative stress. In such conditions, metacaspase expression induced cell death in yeast as well as decreased growth in surviving cells. Furthermore, the CD pathway observed in these MCA-expressing yeast cells was dependent on a z-VAD-fmk inhibitable specific protease. Based on previous results and the outcome of this study, we propose a model of protozoan cell death that places metacaspase as an initiator enzyme activating a downstream effector protease.

## Results

### Expression of PfMCA1-cd-Sc in yeast

**Figure 1:**
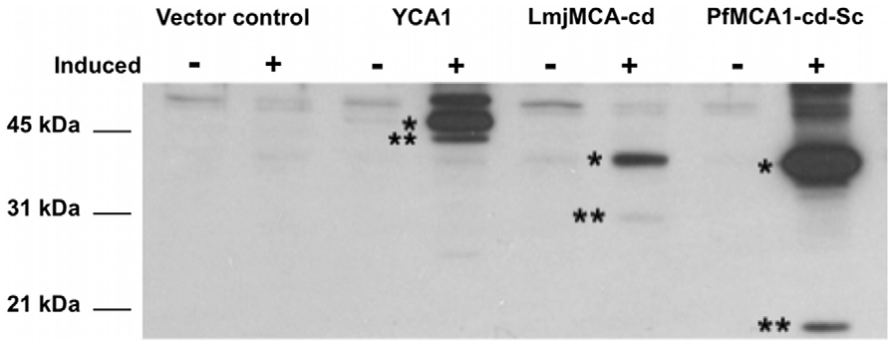
Heterologous expression of PfMCA1-cd-Sc in *Saccharomyces cerevisiae*. *S. cerevisiae* metacaspase 1 (YCA1), the peptidase-C14 domain of the *L. major* metacaspase 1 (LmjMCA-cd) and the optimized nucleic sequence of the peptidase-C14 catalytic domain of PfMCA1 (PfMCA1-cd-Sc) were expressed in yeast for 18 hours with galactose (+) as carbon source. Cells were lysed and 10 µg of the total protein extract was analysed by immunoblotting with anti-M2. For each recombinant protein, the expected molecular mass is represented by a single black star; a double black star represents products of autocatalytic processing.

A chemically synthesized nucleotide sequence coding for the PfMCA1 catalytic domain (PfMCA1-cd-Sc) was designed in accordance with the S. cerevisiae codon usage table and cloned into the pESC-HIS vector with a C-terminal M2-flag. Interestingly, no protein expression was observed by immunoblotting when the natural PfMCA1-cd was used (data not shown), which may have been due to the particular codon usage in P. falciparum [35]. Upon incubation with galactose, PfMCA1-cd-Sc expression was induced in yeast metacaspase null mutants, producing the expected 35kDa polypeptide (theoretical mass of PfMCA1-cd-Sc: 35.55 kDa) that was recognized by anti-M2 antibody (Figure 1, lane 8). Processing was observed for PfMCA1-cd-Sc generating a polypeptide of about 21 kDa (Figure 1, lane 8). Galactose incubation also allowed the expression of two other metacaspases in the Δyca1 cells, i.e the full length YCA1 and the catalytic domain of the L. major metacaspase (LmjMCA-cd). As revealed by immunoblotting using an anti-M2 antibody, two polypeptides of 50 kDa and 35 kDa were detected corresponding to the predicted molecular mass of YCA1 (52.40 kDa) and LmjMCA-cd (36.09 kDa) (Figure 1, lanes 4 and 6). Furthermore, as previously reported [10], [12], YCA1 and LmjMCA-cd were autoprocessed giving rise to two bands of approximately 45 kDa and 31 kDa respectively (Figure 1, lanes 4 and 6). No band was detected in cells transfected with the vector control (Figure 1, lane 2).

### Enzymatic activity of PfMCA1-cd-Sc

**Figure 2:**
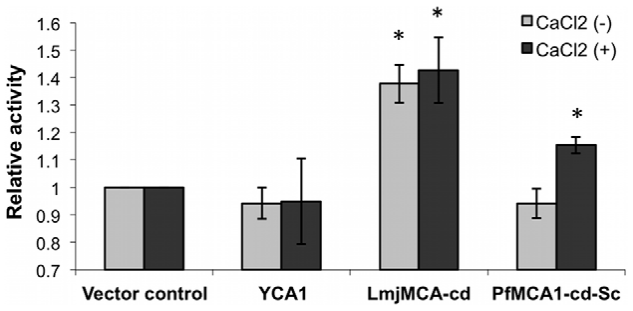
PfMCA1-cd-Sc presents a Ca^2+^-dependent arginine protease activity. *Δyca1* yeasts transfected with the control vector, LmjMCA-cd or PfMCA1-cd-Sc were grown with galactose for 26 hours. 100 µg of total protein extract was tested with the fluorogenic substrates z-VRPR-AMC for 45 min in combination with or without CaCl_2_ (10 mM). Trypsin was used as a positive control of the arginine proteolytic activity (data not shown). Data are represented as the mean ± S.D (n = 3) of the activity relative to the activity present in yeast cells transfected with the vector control. Asterisks (_*_) indicate a significant increase compared to control in the same condition as determined by a Student's *t* test (p<0.05).

We first determined whether PfMCA1-cd-Sc cleaved substrates with an aspartate or arginine in the P1 position. When the caspase specific Ac-DEVD-AMC fluorogenic substrate was used, we failed to detect any significant activity in lysates of cells expressing LmjMCA-cd or PfMCA1-cd-Sc either in the presence or in the absence of calcium whereas a strong aspartate-protease activity (735 mFU/min/µg) could be detected in yeast cells expressing human caspase-6 (data not shown). The MCA arginine-protease specific activity was tested for LmjMCA-cd and PfMCA1-cd-Sc in cell lysates using the fluorogenic substrate z-VRPR-AMC, an optimized substrate for MCAs (Figure 2) [36]. Because it is known that MCA could be calcium dependent in *T. brucei*, *A. thaliana* and *Allomyces arbuscula* [11], [18], [37], [38], [39], we therefore performed our experiments in the absence or presence of calcium. Using a Student's *t* test, we can conclude that LmjMCA-cd showed significant activity for the z-VRPR-AMC substrate compared to the control (p<0.05) (Figure 2). Addition of CaCl2 did not significantly increase its activity. In such assays, presence of calcium does not seem to be essential [40]. In contrast, PfMCA1-cd-Sc was highly calcium dependent, not having any VRPR-specific protease activity in the absence of CaCl2. Indeed, only when calcium concentration was increased to 10 mM, was its activity significant (p<0.05) (Figure 2). These results confirmed that the two protozoan metacaspases harbour a significant activity for substrates having arginine in their P1 position although they could differ in their calcium concentration requirement.

### PfMCA1-cd-Sc induces CD under oxidative stress

**Figure 3:**
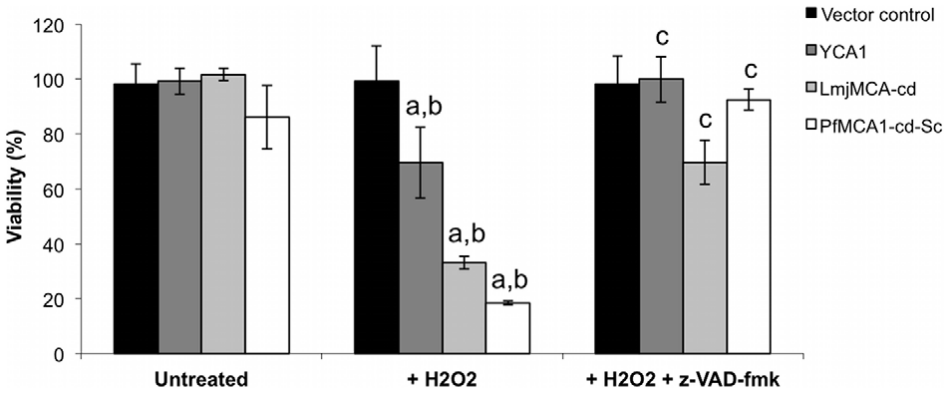
PfMCA1-cd-Sc induces yeast cell death under oxidative stress. *Δyca1* yeast cells transfected with YCA1, LmjMCA-cd, PfMCA1-cd-Sc or the control vector were grown in the presence of galactose and 1 mM H_2_O_2_ for 30 hours with or without inhibitor (z-VAD-fmk, 20 µM). 250 cells were spread on YPG plate and cultured for 48 hours before colony-forming units were counted to estimate cell viability. Data are represented as the mean of cell viability (%)± S.D. (n = 3). **a** indicates a significant difference in the percentage of viability between non treated and H2O2 treated cells as determined by a Tukey's test (p<0.05). **b** indicates a significant difference in viability between MCA expressing lines and the vector control line in the presence of H_2_O_2_ as determined by a Dunnett's test (p<0.05). **c** indicates a significant difference in viability when z-VAD-fmk is present in comparison to conditions with H_2_O_2_ as determined by a Tukey's test (p<0.05).

Autoprocessing of metacaspase polypeptides is required for full activity in the yeast CD pathway induced by oxidative stress [41], [42]. To assess the specific role of the catalytic domain of PfMCA1 in CD we compared the effector function of YCA1, LmjMCA-cd and PfMCA1-cd-Sc under oxidative stress. Transfected yeast cells were exposed to 1 mM H_2_O_2_ and grown in 2% galactose for 30 hours. In order to estimate yeast CD, we performed a cell viability plating assay, an established method of quantifying the effect of MCA on CD, where cell counts are directly linked to cell survival but not to cell proliferation or retarded growth [16], [22]. An equivalent number of cells were plated and colony-forming units (cfu) were counted after 48 hours (Figure 3). We observed significantly different cell viability between the different lines and treatments used as determined by a 2-way ANOVA test (p<0.001 and p<0.001 respectively). As previously reported [10], Δ*yca1* yeast cells transfected with empty vector (pESC-HIS) grew similarly in the absence or presence of H_2_O_2_ (98±8% vs 99±13%) (Figure 3), suggesting that YCA1 expression was required for H_2_O_2_-induced CD in yeast. When YCA1 was expressed in Δ*yca1* yeast cells, we observed a decrease in yeast viability compared to non-treated YCA1 expressing cells or to the control vector in the presence of H_2_O_2_ (70%±8% vs 99%±13%). This decrease was significant as determined by a Student *t* test (p = 0.04) and a Dunnett's test (p = 0.0065) (Figure 3 and Figure S2). In the presence of H_2_O_2_, yeast cell viability was even more affected when expression of LmjMCA-cd or PfMCA1-cd-Sc was induced. We evaluated the survival rate at 33% (±2%) and 18% (±1%) for cells expressing LmjMCA-cd and PfMCA1-cd-Sc respectively (Figure 3). This difference in cell viability observed between *S. cerevisae, Leishmania* and *Plasmodium* metacaspases could be due to the relative amount of the catalytic domain present in the different transfected yeast cells since the survival rate is lower in cells expressing more of the processed catalytic domain. To investigate enzymatic pathways involved in this metacaspase-dependent yeast CD, the pan-caspase inhibitor z-VAD-fmk was added to the yeast growth culture medium. No significant difference was observed in the percentage of viability in cells transfected with the control vector regardless of the presence of H_2_O_2_ or z-VAD-fmk (Figure 3). However, addition of the inhibitor (20 µM) resulted in a significant increase in cell viability in cell lines expressing YCA1, LmjMCA-cd or PfMCA1-cd-Sc (100%±8%, 70±8% and 93±4% respectively, as compared to H_2_O_2_ treated cell and analysed by the Student *t* test, where p<0.01, and by the Tukey's test where p = 0.005, p<0.001and p<0.001 respectively). Taken together, these results show that CD induced by H_2_O_2_ in yeast required expression of MCA but could be rescued by z-VAD-fmk suggesting that this pathway proceeded through MCA and a downstream VAD-fmk sensitive enzyme. Interestingly, although we observed that yeast cells expressing *Plasmodium* or *Leishmania* MCAs responded similarly in the presence of H_2_O_2_ or H_2_O_2_ + z-VAD-fmk as determined by a Tukey's test (p = 0.899), they were significantly more sensitive to H_2_O_2_ than YCA1 expressing yeast as determined by the same statistical analysis (p<0.001). A statistical interaction was found between lines and treatments indicating that they responded differently to the three conditions (2-way ANOVA test; p<0.001). The reason for this variable responsiveness between YCA and protozoan MCA expressing lines is not known but may be due to the relative amounts of the catalytic domain.

### PfMCA1-cd-Sc induces features of late apoptosis in yeast

**Figure 4:**
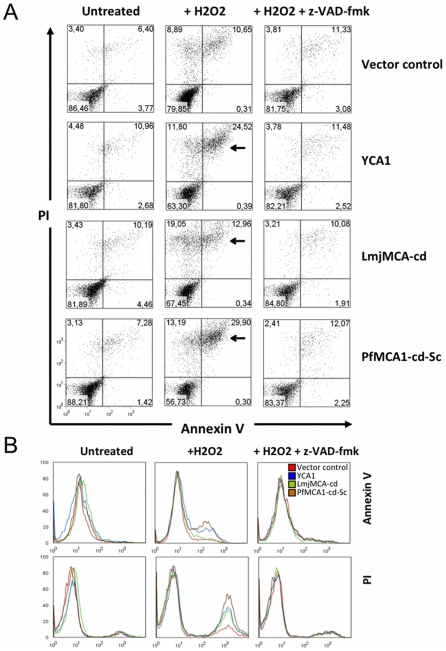
PfMCA1-cd-Sc induces characteristics of late apoptosis in yeast. *Δyca1* yeast cells expressing YCA1, LmjMCA-cd or PfMCA1-cd-Sc were not treated or treated with 1 mM H_2_O_2_ for 33 hours with or without inhibitor (z-VAD-fmk, 20 µM each). Cells were stained with Annexin V (AnV) and propidium iodide (PI). (A) Bivariate flow cytometry analysis of 10000 stained cells. Black arrows indicate dead cells featuring characteristics of late apoptosis (Annexin V positive, PI-positive). Necrotic cells are PI positive and Annexin V negative. (B) Monovariate analysis of cells stained with Annexin V (upper line) or PI (lower line).

To assess whether yeast CD induced by PfMCA1-cd-Sc, LmjMCA-cd or YCA1 under oxidative stress exhibited apoptotic features, we stained the dying cells for the classic markers of apoptosis: propidium iodide (PI) and Annexin V for analysis by FACS. Under normal conditions, Annexin V positive and PI negative cells were not observed for any of the clones (Figure 4A, lane 1) indicating that, at least in the conditions used in our assay, yeast did not exhibit typical early apoptotic markers. By contrast, a significant population of cells expressing markers of late apoptosis (e.g. Annexin V/PI double positive cells) were observed after the addition of H_2_O_2_ in yeast cells expressing YCA1 or PfMCA1-cd-Sc (Figure 4A lane 2). Here, the double positive population increased from 11% under normal conditions to 24% when YCA1 complemented Δ*yca1* cells were grown under oxidative stress. Similarly, for yeast expressing PfMCA1-cd-Sc, we found a 30% double positive population. Analysis revealed no significant difference in this staining for yeast cells transfected with LmjMCA-cd DNA in the presence or in the absence of H_2_O_2_ as was the case when cells were transfected by a plasmid lacking MCA used as negative control. However, a monovariant analysis showed that PI labelling was significantly higher for yeast cells expressing LmjMCA-cd in the presence of H_2_O_2_ as determined by a Student's *t* test (p<0.05) (Figure 4B). To determine whether late apoptosis markers were related to a VAD dependent pathway, we added the z-VAD-fmk inhibitor (20 µM final) in H_2_O_2_-stressed culture. In such conditions, we observed 11% and 12% of Annexin V/PI positive cells for YCA1 and PfMCA1-cd-Sc respectively. These percentages were similar to those in the absence of oxidative stress (11% and 7%) confirming that a VAD inhibitor can rescue cells and thus abrogates the effect of metacaspase. In conclusion, these data indicate that YCA1, LmjMCA-cd and PfMCA1-cd-Sc expression in H_2_O_2_ stressed *Δyca1* yeast induces a hallmark of late apoptosis in a VAD-dependent manner.

### Effect of PfMCA1-cd-Sc expression on yeast growth

**Figure 5:**
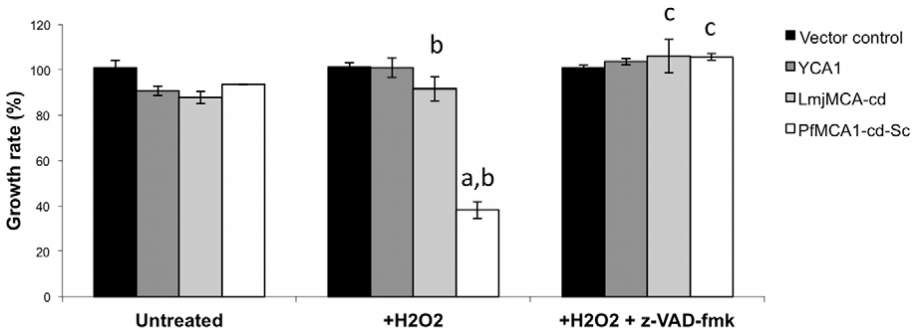
PfMCA1-cd-Sc retards yeast growth under oxidative stress. YCA1, LmjMCA-cd and PfMCA1-cd-Sc were expressed in *Δyca1* cells with H_2_O_2_ (1 mM) and with or without inhibitor (z-VAD-fmK, 20 µM). Transfected yeasts were grown for 30 hours before yeast proliferation was determined by the MTS proliferation assay. MTS activity was measured by spectrophotometry at 492 nm every 2 hours and the growth rate was determined as described in methods. Data are represented as the mean ± S.D. (n = 3). **a** indicates a significant difference in the percentage of viability between non treated and H_2_O_2_ treated cells as determined by a Tukey's test (p<0.05). **b** indicates a significant difference in viability between MCA expressing lines and the vector control line in the presence of H_2_O_2_ as determined by a Dunnett's test (p<0.05). **c** indicates a significant difference in viability when z-VAD-fmk is present in comparison to conditions with H_2_O_2_ as determined by a Tukey's test (p<0.05).

The involvement of metacaspase proteins in the retardation of cell growth has been previously reported [43], [44] and led us to investigate whether CD mediated by PfMCA1-cd-Sc was associated with a dysregulation of cell cycle progression. The growth rate, as measured by an MTS assay, was determined every 2 hours in liquid culture (Figure 5). We observed a significant difference in growth rate between the lines and treatments used as determined by a 2-way ANOVA test (p<0.001 and p<0.001 respectively). Under oxidative stress, the growth rate of PfMCA1-cd-SC expressing cells was significantly reduced compared to either non -treated PfMCA1-cd-SC expressing cells (38%±4% versus 93%) or to H_2_O_2_ treated cells transfected with the vector alone (100%), as determined by the Student *t* test (p<0.01) or by the Tukey's or Dunnett's tests respectively (p<0.001 for both). We observed a small but significant decrease in growth rate for yeast expressing LmjMCA-cd when treated with H_2_O_2_ in comparison to cells transfected with the vector alone (p = 0.045). However, no difference was observed when growth was compared to untreated LmjMCA-cd expressing controls (p = 0.692). Finally, no significant effect on yeast cell cycle progression was observed in YCA1 complemented cells. Differences observed between PfMCA1-cd-Sc and the two other MCAs was certainly due to the lower level of expression of these polypeptides (Figure 1) in the complemented yeast as compared to PfMCA1-cd-Sc. The addition of z-VAD-fmk significantly abolished the effect of PfMCA1-cd-Sc as determined by the Student *t* test (p<0.01) suggesting that the two enzymes, i.e. metacaspase and z-VAD-fmk sensitive enzyme, could be part of the same pathway.

**Figure 6:**
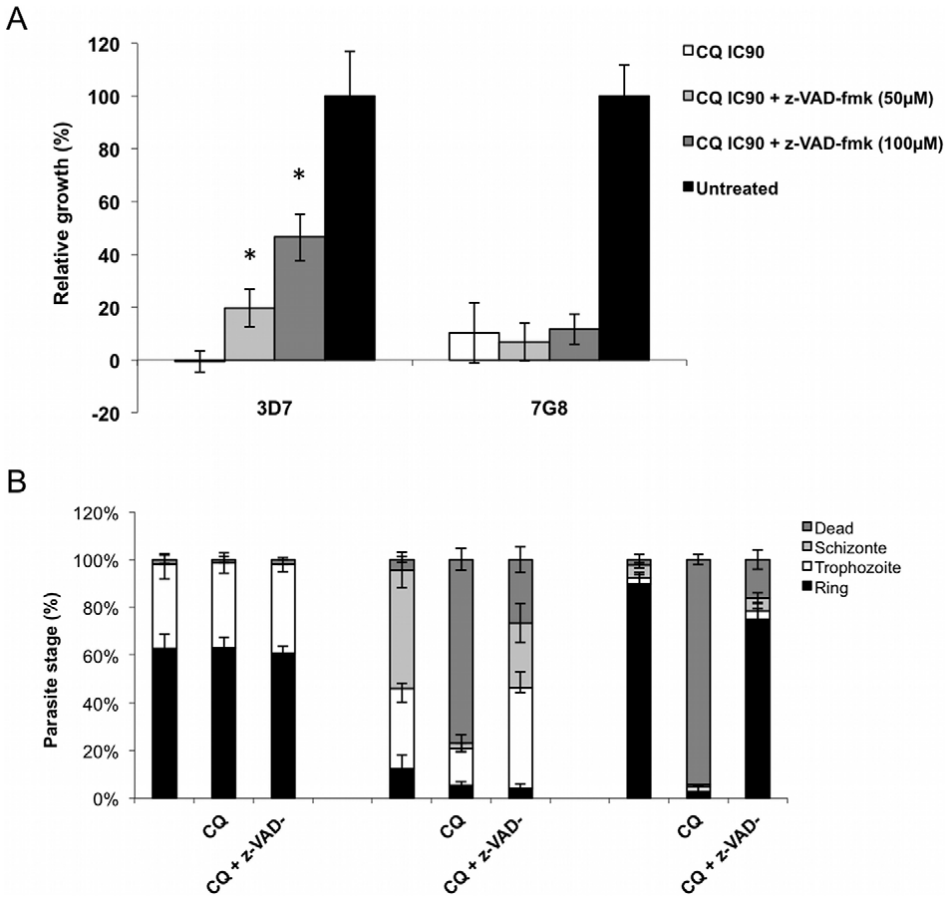
Z-VAD-fmk partially abrogates cell death and growth inhibition induced by CQ in *Plasmodium falciparum* parasites. (A) A CQ sensitive clone (3D7) and a CQ resistant clone (7G8) were cultured with or without CQ to a final concentration corresponding to IC_90_ in presence or absence of z-VAD-fmk (50 µM or 100 µM final concentration). Parasitemia was evaluated by Giemsa staining at 6, 24 and 44 hours and the relative growth compared to untreated parasites was determined. Data are represented as the mean ± S.D. (n = 3). Asterisks indicate a significant increasing of parasitemia compared to cultures treated with CQ without inhibitor (Student's test * = p<0.01). (B) Percentage of each maturation forms observed for a 3D7 *P. falciparum* culture at 6 hours, 24 hours and 48 hours following CQ addition (at final concentration corresponding to IC_90_) with or without z-VAD-fmk (100 µM). Data are represented as the mean ± S.D. (n = 3).

Involvement of a VAD-protease pathway in yeast expressing PfMCA1-cd-Sc led us to consider whether a similar pathway might be involved in *P. falciparum* proliferation under antimalarial drug pressure (Figure 6). We performed our experiment using erythrocytic stages of *P. falciparum*, as most of the available antimalarial drugs target this stage *in vivo*; here, we evaluated the effect of z-VAD on maturation of chloroquine-treated parasites by culturing two different *P. falciparum* clones in the presence or in the absence of z-VAD-fmk: clone 3D7 which is sensitive to chloroquine (IC_50_ = 23±11 nM) and 7G8 resistant to it (IC_50_ = 161±55 nM). We then measured the effect on parasitemia and parasite maturation at 6, 24 and 44 hours after incubation with the respective IC_90_ chloroquine concentrations (IC_90_ = 81±28 nM for 3D7 and IC_90_ = 679±260 nM for 7G8). As expected, 3D7 and 7G8 parasites were killed at their respective IC_90_. However, after addition of z-VAD-fmk to a final concentration of 50 µM or 100 µM, a growth rate of 20% and 46% were obtained in the untreated, CQ-sensitive control (Figure 6A) while no effect on proliferation or maturation was observed in the CQ resistant clone. Determination of the different maturation forms indicated that 3D7 parasites were able to complete their erythrocytic cycle when the z-VAD-fmk inhibitor was added to CQ treated cultures (Figure 6B). Further, CQ sensitivity of these clones was reduced in the presence of 100 µM of z-VAD-fmk (Table 1) where the IC_90_ value significantly increased for the susceptible 3D7 clone (from 81±28 nM to 560±67 nM; p<0.05) while no significance was found for CQ resistant 7G8 clones in either IC_50_ or IC_90_ value (Table 1). These data support the hypothesis that the CD pathway induced by chloroquine stress in *P. falciparum* is dependant on a z-VAD-fmk inhibitable protease.

**Table 1:** z-VAD-fmk decreases 3D7 *P. falciparum* CQ sensitivity.

	3D7			7G8		
	IC_50_	IC_90_	IC_95_	IC_50_	IC_90_	IC_95_
CQ	23±11	81±28	107±24	161±55	679±260	792±275
CQ +50 µM z-VAD-fmk	206±50*	646±68*	745±70*	363±117	1095±68	1289±97
CQ +100 µM z-VAD-fmk	161±32*	560±67*	720±13*	266±96	898±205	1077±185
CQ +0.05% DMSO	16±3	58±23	84±36	172±12	488±69	582±98
CQ +0.1% DMSO	35±16	94±28	112±22	177±32	476±54	561±57

*P. falciparum* CQ sensitive (3D7) or resistant (7G8) clones were cultured under a graded concentration of CQ in combination with 50 µM or 100 µM z-VAD-fmk. IC_50_, IC_90_ and IC_95_ were determined using the SYBR Green I fluorescence method. Use of pure DMSO at a similar percentage to that created by the addition of z-VAD-fmk acted as a control. Data are represented as the mean S.D. (n = 3). Asterisks indicate a significant increase of IC compared to IC obtained without inhibitor (* = p<0.05).

## Discussion

In this study, we have performed a comparative functional analysis of MCA from three different organisms, *S. cerevisiae*, *P. falciparum* and *L. major* and provided evidence for a CD pathway implicating MCA and a VAD-binding enzyme. In PfMCA1-cd-Sc-expressing yeast, we observed 82% cell death after oxidative stress compared with 30% and 67 % in cells expressing YCA1 and LmjMCA-cd, respectively. It is likely that this increased sensitivity is the consequence of higher levels of expression of the *P. falciparum* catalytic domain in comparison to YCA1 and LmjMCA-cd. These results are in agreement with our previous observations that 1) CD is dependent on the expression of an MCA catalytic domain and 2) the catalytic domain is more efficient in inducing CD than the full-length protein, which requires processing to gain complete activity [12], [15].

Morphology does not clearly delineate the boundaries between necrosis, apoptosis, necroapoptosis or other CD outcomes [45] and thus, dead cells were identified by membrane disruption (PI staining). After H_2_O_2_ treatment, 43%, 36% and 32% of the cells were PI positive in PfMCA1-cd-Sc, YCA1 and LmjMCA-cd-expressing populations, respectively (Figure 4A). Double staining of PI and Annexin V is indicative of necrosis or late apoptosis; a phenotype that has been predominant in several similar CD studies on yeast [16], [46]. Interestingly, in our experimental design, PI staining decreased in the presence of z-VAD-fmk, similarly to cathepsin-B like deficient *Leishmania* parasites [23] suggesting that necrosis-like CD can be suppressed by treatment with specific inhibitors or in genetically deficient parasites lacking lysosomal enzymes such as cathepsin-B, which can bind z-VAD-fmk [23]. Although we cannot definitively conclude whether CD in protozoan parasites is necrotic or apoptotic, our data support a CD mechanism that is under genetic control harbouring both necrotic and apoptotic features. Further investigation based on Annexin V and PI staining would help distinguish between necrosis and apoptosis when samples are analysed at different time points in the presence of specific inhibitors. Specifically, analysing the nuclear translocation of endonuclease G [47], [48] or inhibition of apoptosis by overexpression of Bcl-XL [49], [50] may allow us to better differentiate between the two processes.

*P. falciparum* erythrocytic stages are the major targets of most available antimalarial drugs. We have previously shown that parasites died under chloroquine (CQ) pressure, forming pyknotic parasites in a VAD-protease dependent manner [13], [51]. This caspase-like CD was specific to *P. falciparum* killed by CQ. Here, we confirmed these data showing that z-VAD-fmk restores proliferation under CQ pressure and reduces the CQ sensitivity (Figure 6). We can speculate that CQ sensitivity is increased in cell death competent parasites but can be decreased by specific inhibition. This point provides preliminary evidence that pro-apoptotic or -necrotic partner drugs could be tested alongside standard anti-malarial drugs in order to increase or restore their efficacy in instances of multi-resistance.

Cell death is prevented when MCA is absent or after z-VAD-fmk treatment in *Plasmodium* and in MCA over-expressing yeast cells (Figure 3 and Figure 4). This suggests that two different proteolytic activities are implicated in the cell death pathway, i.e. MCA and a VAD-binding enzyme. In plant embryogenesis, the CD pathway requires a caspase-like protease activity where the executioner, AtMCA2, harbours an arginine-specific activity essential for CD [14], [52], [53]. Apoptotic features in *T. cruzi,* induced by FHS-treatment have been associated with an increased YVADase activity [54], whereas VADase is attributed to the phenotype seen in *P. berghei* and *P. falciparum* when induced by CQ [55], [56]. However, protozoan parasites do not code for caspase but rather metacaspases, members of an arginine and/or lysine specific protease family [10], [12], [37], [40], which cannot account for the caspase-like activity. Thus, CD induced by oxidative stress is likely to be mediated by metacaspase with arginine-specific proteolytic activity in a VAD-dependent pathway. Such a pathway could also be present in plants and in unicellular organisms.

**Figure 7:**
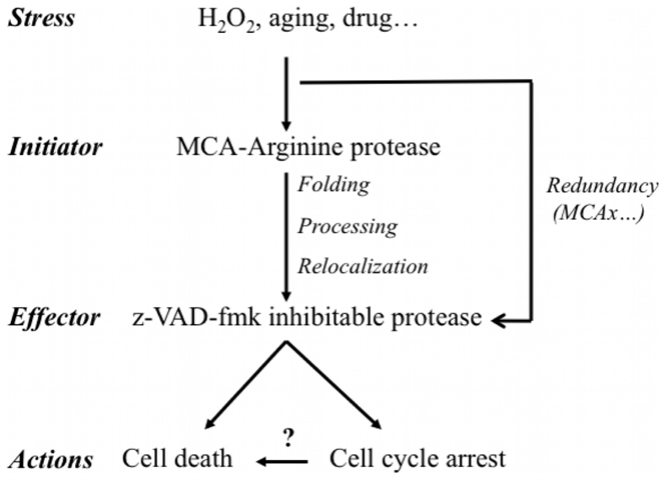
Metacaspase proteins in cell death and cell growth pathways. Metacaspase proteins could play a key role in the balance between life and death at a cellular level. Here they are predicted as the initiator proteins inducing cell death through a z-VAD-fmk inhibitable effector protease. The wide range of metacaspase functions suggests this activation could occur through 1) protein relocalization, 2) remodelling, or 3) specific processing mediated by an arginine-specific proteolytic activity. A secondary pathway could occur via the activation of other (meta)caspase proteins (MCAx) leading to a functionally redundant pathway.

Although we have yet not identified the z-VAD-fmk binding enzyme in *Plasmodium* as reported in *Leishmania* [23], nor provided evidence that MCA acted directly on a VAD-binding enzyme (i.e. cathepsin B released from its cellular compartment), our results on VAD-fmk counteracting the MCA effect lead us to propose a general model in which metacaspase is placed upstream of a VAD-fmk inhibited protease. Activated by stress agents such as H_2_O_2_, metacaspase is processed into an active catalytic domain that could be involved in activating a downstream protease. This latter enzyme may function as the final effector of CD. Thus, we complete the protease cascade model of Ch'ng et al. [20], proposing that the CD pathway is mediated by MCA, which consequently activates clan CA cysteine proteases, such as cathepsins. This is linked to downstream mitochondrial outer membrane permeabilization (MOMP) and an additional release of CA protease that is amplified by mitochondrial dysfunction and DNA degradation (Figure 7). Placing MCA in an upstream position in the CD cascade could explain why, in some reports, no prominent effect on cell death is observed in metacaspase deficient organisms [57], [58], [59] or that a relevant loss-of- function phenotype was not observed in metacaspase gene silencing or disruption of parasite MCA alleles, suggesting that either 1) a redundant CD signalling pathway exists in parasites, 2) the CD induction is able to by-pass MCA action [58] or, 3) as was shown in *A. thaliana*, different allelic MCA forms can regulate each other complicating the characterization of the CD pathway [14].

Recent reports have highlighted the diversity of metacaspase functions possibly related to the ancient origin of this family [60] and not restricted to CD [7], [8], [61]. Some metacaspases seem to play a central role in cell growth, for example, overexpression of *T. brucei* metacaspase 4 (TbMCA4) in budding yeast resulted in growth inhibition compared to wild type cells [19] while triple RNAi of MCA2, MCA3 and MCA5 in *T. brucei* led to total growth arrest [57]. In *Leishmania*, overexpression of the single metacaspase LmjMCA induced growth retardation and alteration of cytokinesis [44]. Paradoxically, overexpression of the *S. pombe* metacaspase 1 (Pca1^+^) stimulated cell growth [62] and it was shown that the metacaspase found in the primitive chytridiomycete fungus, *Allomyces arbuscula*, may promote the cell cycle, as it is upregulated during the vegetative growth phase [39]. This inspired us to explore the role of PfMCA1-cd-Sc in the cell cycle and interestingly, we observed a decrease of the growth rate from 93% for the vector control to 38% for yeast expressing the catalytic domain of *P. falciparum*. These data confirm the catalytic role of PfMCA1-cd-Sc in CD and in cell growth retardation, but whether these two functions are related and whether cell growth retardation is a necessary step of CD requires further investigation.

The interest for adjunctive therapy, such as the use of erythropoietin in children suffering of cerebral malaria is now increasing [51], and it is conceivable that activation of parasite metacaspases and/or of the downstream VAD-binding enzyme may help increase the speed of parasite clearance as well as the efficiency of anti-parasitic drugs. For such a therapeutic approach to be valuable, a better understanding of how and why parasites and unicellular organisms undergo CD is of absolute importance.

## Methods

### DNA constructs, protein expression and immunodetection

To obtain expression in *S. cerevisiae*, we optimized the DNA sequence encoding the PfMCA1 catalytic domain (amino acid residues 287 to 573). This was done according to the *S. cerevisiae* codon usage tables based on yeast coding sequences (codon usage table Eyeast.cut) or on highly expressed genes (codon usage table Eysc_h.cut; http://www.kazusa.or.jp/codon/). Modifications were performed to obtain similar percentage of *S. cerevisiae* codon usage and GC percentage. Additionally 3′end signals and poly(A) sites were removed and these optimized nucleic sequences are described in Figure S1. The probability of heterologous expression of PfMCA1-cd-Sc in yeast was evaluated with the codon usage adaptation index factor (CAI factor) and a value of 0.808 was obtained indicating a high chance of protein expression in *S. cerevisiae*. Similarly, GC percentage was improved from 26.8% for the native gene to 37.2% for the optimized one, a value similar to the GC percentage of the *S. cerevisiae* coding sequences (39.77%). 

A consensus Kozak sequence (5′-GCCGCCACC-3′) was added upstream to the initiator codon of PfMCA1-cd-Sc. Two restriction sites, EcoR1 and Not1, were added in 5′ and 3′ of the construct. The optimized nucleic PfMCA1-cd-Sc sequence was synthetized chemically and inserted into the pUC57 vector by the GeneCust Company (GeneCust, Dudelang, Luxembourg). Competent Top10 cells were transformed with the PfMCA1-cd-Sc-pUC57 plasmid, which then was digested with EcoR1 and Not1. This was cloned into the pESC-HIS vector in frame with the M2-FLAG (Stratagene; La Jolla, CA, USA) to generate a construct (pESC-PfMCA1-cd-Sc) coding for a C-terminal FLAG-tagged PfMCA1-cd-Sc protein under the control of the GAL10 promoter. The complete PfMCA1-cd-Sc construct was sequenced on both DNA strands (Genome Express, Meylan, France). The pESC-HIS DNA constructs encoding a full length C-terminal M2-tagged yeast metacaspase 1 (pESC-YCA1) as well as the catalytic domain of *L. major* metacaspase 1 (pESC-LmjMCA-cd) were described previously [12]. The Euroscarf *YCA1* disrupted strain (Δ*yca1* yeast cells) [Accession No. Y02453 (BY4741; MAT a; his3Δ1; leu2Δ0; met15Δ0; ura3Δ0; YOR197w::kanMX4)] was transfected with the constructs pESC-HIS, pESC-YCA1, pESC-LmjMCA-cd and pESC-PfMCA1-cd-Sc respectively.

### Yeast culture and protein expression

The YCA1 deficient yeast was transfected with the constructs pESC-HIS, pESC-YCA1, pESC-LmjMCA-cd and pESC-PfMCA1-cd-Sc respectively. Transfected yeast cells were selected and grown in synthetic/dropout (SD/DO) culture medium consisting of yeast nitrogen base (6,7 g/L, Becton, Dickinson and Company, Sparks, MD), a dropout amino acid solution without histidine (20 mg/L adenine hemisulfate salt, arginine monohydrochloride, methionine, tryptophan and uracil; 30 mg/L isoleucine, lysine monohydrochlorid and tyrosine; 50 mg/L phenylalanine; 100 mg/L leucine; 150 mg/L valine and 200 mg/L threonine), geneticin (200 mg/L, Invitrogen, Carlsbad, CA, USA) and 2% glucose. Cells were grown on SD/DO plates with 2% agar (Becton, Dickinson and Company) and plates incubated at 30°C for 4 days. Ten millilitres of SD/DO non-inducing selective medium were inoculated with one colony and incubated overnight at 30°C with continuous shaking. Cultures were diluted to an OD_600_ of 0.05 in 10 ml SD/DO non-inducing selective medium and kept in culture until reaching an OD_600_ of approximately 0.5. Cells were centrifuged and diluted in 1 vol. of SD/DO medium containing 2% galactose instead of glucose for induction of protein expression for 18 hours. Cells were centrifuged and the pellets were kept frozen at −80°C before use.

### Immunodetection

Frozen pellets were resuspended in 150 µL of lysis buffer (50 mM KH2PO4 pH 7.5, 500 mM NaCl, 1 mM EDTA, 5 mM DTT, 1% vol CHAPS) and vortexed ten times in the presence of 0.08 g of 0.25 mm glass beads. Quantification of total protein was performed by BCA (Thermo Scientific Pierce, Rockford, IL, USA). Total protein (10 µg) was loaded and separated on a 12% polyacrylamide gel and electrophoretically transferred to a nitrocellulose membrane (Immobilon-P; Millipore, Billerica, MA, USA). Membranes were blocked with 5% (w/v) milk in TBST buffer (25 mM Tris-HCL, pH 7.4, 140 mM NaCl, 0.1 (v/v) Tween 20) for 1 hour at RT. The M2 epitope was detected using the commercially available anti-M2 antibody (1∶2000 dilution, Cat 200471, Stratagene, La Jolla, CA, USA) in TBST buffer with 1% (w/v) milk for 1 hour at RT. Membranes were washed in TBST buffer and incubated with an anti-mouse IgG horseradish peroxidase–conjugated secondary antibody (1∶2500 dilution, Cat: W4021, Promega, Madison, WI, USA) for 1 hour at RT with 1% (w/v) milk in TBST buffer. The immunostained proteins were visualized with enhanced chemiluminescence (Lumi-Light Western blotting substrate, and Lumi-Film, Roche Applied Science, Indianapolis, IN, USA).

### Survival test

The effect of oxidative stress on yeast expressing metacaspase proteins was quantified by a plating assay. Transfected yeast cells were grown for 30 hours in 2% galactose containing SD/DO culture medium with or without H_2_O_2_ (1 mM final concentration, Sigma-Aldrich, Saint Louis, MO, USA) and in the presence or absence of 20 µM final concentration of z-VAD-fmk protease inhibitor. After cell counting in a Neubauer chamber, 250 cells were plated on a non selective solid YPD culture medium consisting in 20 g/L Difco peptone (Merk, Darmstadt, Germany), 10 g/L yeast extract (Becton, Dickinson and Company, Sparks, MD, USA), 20 g/L agar (Becton, Dickinson and Company, Sparks, MD, USA), and 2% glucose. Yeast cells were grown for 48 hours at 30°C and colonies were counted. Assays were performed in duplicate in three independent experiments.

### Proliferation test

Yeast growth rate and proliferation were assessed by the CellTiter 96 Aqueous Non-Radioactive Cell Proliferation assay (MTS assay; Promega, Madison, Wisconsi, USA). This was performed 30 hours post induction concomitantly with the survival test. Briefly, once protein expression had been induced, the same number of cells was added to 96 well plates (100 µL at OD_600_ = 0.5) with 20 µL of the combined MTS/PMS solution. Plates were incubated at 30°C with continuous agitation then absorbance was measured at 492 nm every second hour during a 12 hours period. Growth rate (GR) was calculated according to the number of generations that developed per unit of time in an exponentially growing culture between 4 and 8 hours, and therefore calculated as GRfln(OD_492_ at 8 hours-OD_492_ at 4 hours)/4.

### Cell death markers

Annexin V (AnV) and propidium iodide (PI) labelling were used to investigate yeast apoptotic features. Cells were exposed to different conditions for 33 hours as previously described in the survival test. For each condition, 10^7^ cells were washed in 50 µL sorbitol buffer (1.2 M sorbitol, 0.5 mM MgCl2, 35 mM KH2PO4, pH 6.8), resuspended in 50 µL Tris/DTT buffer (100 mM Tris pH 9.4, 10 mM DTT), washed in the sorbitol buffer before being incubated for 1.5 hour under continuous agitation at 30°C with 50 µL Zymolyase solution (Zymolyase 20 T, Seikagaku Corp., Tokyo, Japan, 1 mg/mL in sorbitol buffer). Cells were centrifuged, resuspended in 50 µL binding buffer and 5 µL Annexin V was added for 20 min at RT in the dark. Cells were centrifuged again and the pellet was diluted in 250 µL binding buffer with PI (10 µg/µL final concentration). Fluorescence analysis of 10000 cells was performed with a BD FACScan™ apparatus. Data were analysed using CellQuest™ (Becton-Dickinson Bioscience, San Jose, CA, USA) and FlowJo™ V.7.2.5 software (Tree Stra Inc., Ashland, OR, USA).

### Enzymatic activity

To investigate metacaspase protease activity, two fluorogenic substrates z-DEVD-AMC (Biomol International LP, Plymouth Meeting, PA, USA) and z-VRPR-AMC [36] were used. From total protein extract for immunodetection, 100 µg of protein was diluted in 200 µL activity buffer (150 mM NaCl, 25 mM HEPES, 10% glycerol, 0.1% CHAPS, 10 mM DTT) containing z-DEVD-AMC or z-VRPR-AMC at 50 µM. Additionally, 10 mM final concentration of CaCl_2_ was added to the activity buffer. The amount of AMC liberated during the reaction was measured fluorimetrically each 15 min during a 45 min period at RT (excitation: 355 nm; emission: 460 nm). Protease activity assays were performed in duplicate, in three independent experiments.

### *In vitro* parasite cultivation and inhibitory concentration (IC) determination

Asexual *P. falciparum* cultures of reference clone 3D7 (chloroquine sensitive; obtained from ATCC/MR4) and 7G8 (chloroquine resistant; obtained from ATCC/MR4) were maintained in culture by standard methods. 3D7 and 7G8 clones of *P. falciparum* were cultivated using O^+^ human erythrocytes at 5% hematocrit in RPMI 1640 medium with phenol red (Invitrogen, Carlsbad, CA, USA) supplemented with 24 mM sodium bicarbonate, 35 mM HEPES buffer, 10 µg/mL gentamycin, and 0.005 g/L albumax. Parasitized red blood cells (pRBCs) were maintained as thin layers at 37°C in an environment containing 5% O_2,_ 5% CO_2_ and 90% N_2_ on a 24 hours medium-change schedule. Parasite growth was determined as the percentage of infected erythrocytes (parasitemia) monitored by observation of Giemsa-stained smears. Determination of the 50, 90 and 95% inhibitory concentrations (IC_50_, IC_90_, IC_95_) was performed using an assay based on the incorporation of the SYBR Green I molecule, a fluorescent DNA double-strand dye [63]. Cultures were diluted to reduce parasitemia to 0.5%, and hematocrit to 1.5% with fresh human RBCs. A total of 175 µl/well was added in duplicate to a 96-well plate containing 25 µl of chloroquine diphosphate (Sigma-Aldrich, Inc, St-Louis), from 0 nM to 1600 nM final concentration with or without z-VAD-fmk (50 µM or 100 µM final concentration). Following a 72 hour incubation period, the plates were frozen and stored at −80°C until the SYBR green I assay. The plates were thawed at RT and 100 µl of the culture was transferred to a new 96-well plate, followed by the addition of 100 µl of SYBR green I (Molecular Probes, Invitrogen, Carlsbad, CA) in lysis buffer (0.2 µl of SYBR green I/ml of 2×lysis buffer, which consisted of 20 mM Tris, 5 mM EDTA, 0,008% (wt/vol) saponin and 0.08% (wt/vol) Triton X-100). The plates were covered and incubated at RT for 1 hour. The fluorescence intensity was measured with a GENius Plus plate reader (Tecan USA, Research Triangle, NC) (excitation: 485 nm; emission: 535 nm; gain set: 60). The IC_50_, IC_90_ and IC_95_ obtained after incubation were calculated by using the HN-NonLin V1-1 software.

### Statistical analysis

Minitab software (version 15.1) was used for the statistical analysis. Before evaluation, normal distribution of data and equal variance were verified with the Anderson-Darling and the Bartlett's test respectively. The impact of the different treatments was tested in a one- or 2-way ANOVA when data were normally distributed. Paired comparisons were performed using Student *t*'s, Tukey's test or Dunett's testings. Results are represented as mean of 3 independent experiments +/- SD values. Differences were considered significant at p<0.05 or highly significant at p<0.01.

## Supporting Information

Figure S1 **Nucleic sequences of the native PfMCA1 peptidase-C14 domain (PfMCA1-cd, A) and the optimized PfMCA1-cd-Sc (B).**
(TIF)

Figure S2 **PfMCA1-cd-Sc induces yeast cell death under oxidative stress.** Δ*yca1* transfected yeasts with PfMCA1-cd-Sc, YCA1, LmjMCA-cd or vector control were grown with galactose and 1mM H_2_O_2_ for 30 hours with or without inhibitor (z-VAD-fmk 20 µM). 250 cells were spread on YPG plate and cultured for 2 days. Pictures show colony-forming units before cell viability was estimated. Pictures are representative of three independent experiments. Plate diameter is 100 mm.
(TIF)
