# Supplementary figures and images for: Plasmodium falciparum Metacaspase PfMCA-1 Triggers a z-VAD-fmk Inhibitable Protease to Promote Cell Death
Source: PLoS One. 2011 Aug 17;6(8):e23867. doi: 10.1371/journal.pone.0023867 (PMC3157471; doi:10.1371/journal.pone.0023867)

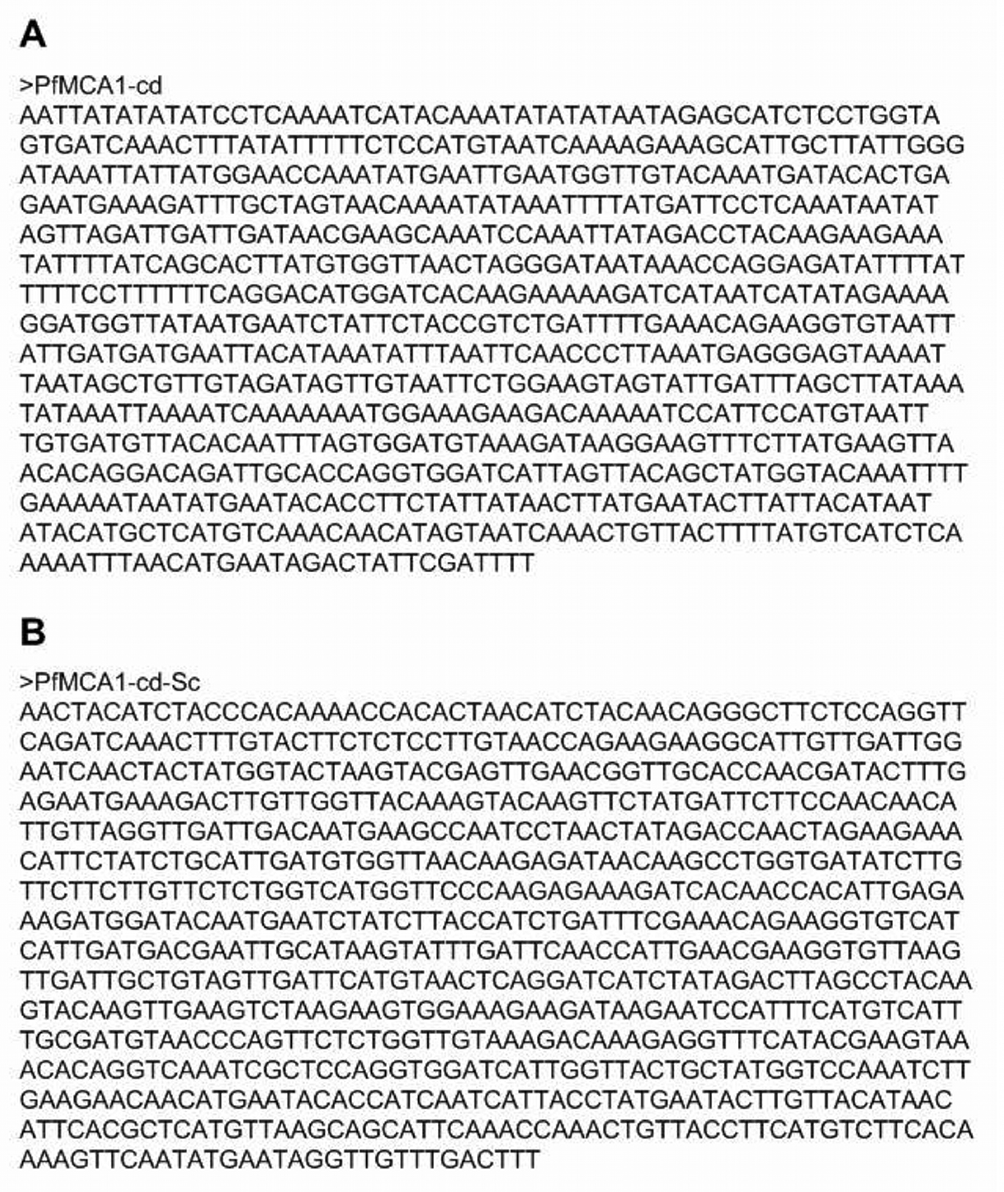

Supplement: Figure S1 — Nucleic sequences of the native PfMCA1 peptidase-C14 domain (PfMCA1-cd, A) and the optimized PfMCA1-cd-Sc (B). (TIF) [file pone.0023867.s001.tif]

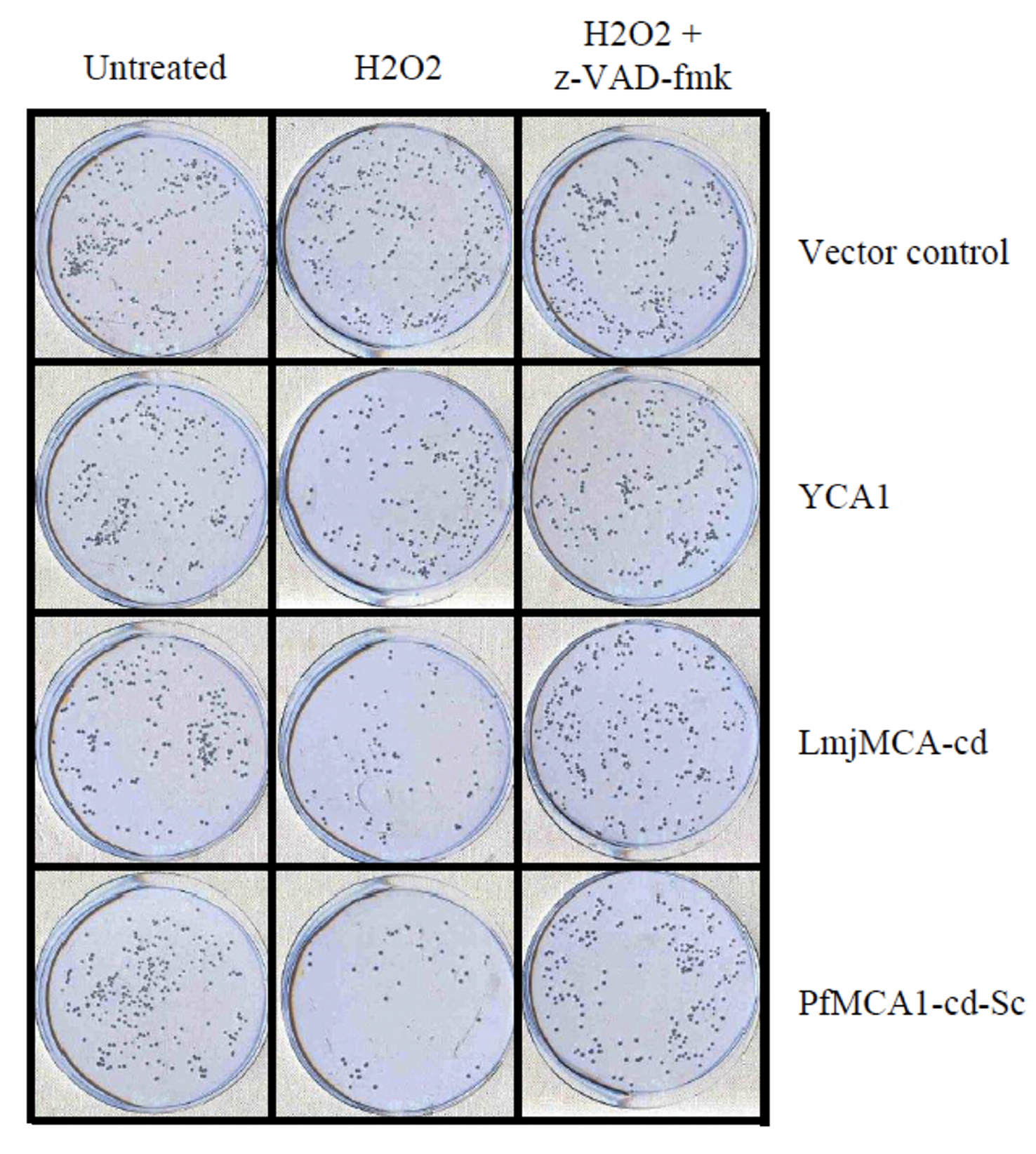

Supplement: Figure S2 — PfMCA1-cd-Sc induces yeast cell death under oxidative stress. Δyca1 transfected yeasts with PfMCA1-cd-Sc, YCA1, LmjMCA-cd or vector control were grown with galactose and 1mM H2O2 for 30 hours with or without inhibitor (z-VAD-fmk 20 µM). 250 cells were spread on YPG plate and cultured for 2 days. Pictures show colony-forming units before cell viability was estimated. Pictures are representative of three independent experiments. Plate diameter is 100 mm. (TIF) [file pone.0023867.s002.tif]
